# Supplementary material for: Optimal n-Type Al-Doped ZnO Overlayers for Charge Transport Enhancement in p-Type Cu2O Photocathodes
Source: Micromachines (Basel). 2021 Mar 22;12(3):338. doi: 10.3390/mi12030338 (PMC8004703; doi:10.3390/mi12030338)
Supplement: Supplementary file 1 [file micromachines-12-00338-s001.pdf]

micromachines

Article

Optimal n-Type Al-Doped ZnO Overlayers for Charge Transport Enhancement in p-type Cu_2O Photocathodes

Hak Hyeon Lee [1,†], Dong Su Kim [1,†], Ji Hoon Choi [1], Young Been Kim [1], Sung Hyeon Jung [1], Swagotom Sarker [2], Nishad G. Deshpande [2], Hee Won Suh [1] and Hyung Koun Cho [1,*]

1 School of Advanced Materials Science and Engineering, Sungkyunkwan University, 2066 Seobu-ro, Jangan-gu, Suwon, Gyeonggi-do 16419, Korea; zadxs@skku.edu (H.H.L.); dskim2846@naver.com (D.S.K.); Jihoon9290@skku.edu (J.H.C.); yb3201@skku.edu (Y.B.K.); wjdtjdgus2@skku.edu (S.H.J.); naekkeo@skku.edu (H.W.S.)
2 Research Center for Advanced Materials Technology, Sungkyunkwan University, 2066 Seobu-ro, Jangan-gu, Suwon, Gyeonggi-do 16419, Korea; nicedeshpande@gmail.com (N.G.D.); swagotom@yahoo.com (S.S.)
* Correspondence: chohk@skku.edu (H.K.C.)
† These authors contributed equally to this article.

Citation: Lee, H.H.; Kim, D.S.; Choi, J.H.; Kim, Y.B.; Jung, S.H.; Sarker, S.; Deshpande, N.G.; Suh, H.W.; Cho, H.K. Optimal n-Type Al-Doped ZnO Overlayers for Charge Transport Enhancement in p-type Cu_2O Photocathodes. *Micromachines* **2021**, *12*, x. https://doi.org/10.3390/xxxxx

Academic Editor: Fabio Di Pietrantonio

Received: 23 February 2021
Accepted: 17 March 2021
Published: 22 March 2021

Publisher's Note: MDPI stays neutral with regard to jurisdictional claims in published maps and institutional affiliations.

Copyright: © 2021 by the authors. Licensee MDPI, Basel, Switzerland. This article is an open access article distributed under the terms and conditions of the Creative Commons Attribution (CC BY) license (http://creativecommons.org/licenses/by/4.0/).

Table S1. Band gap of prepared overlayers estimated by Tauc plot.

Sample	Band gap (eV)
ZnO	3.22
AZ@AZO	3.31
AA@AZO	3.33
ZA@AZO	3.29
ZZ@AZO	3.32

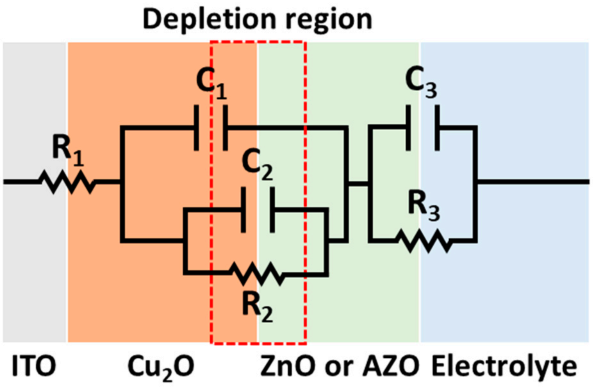

Figure S1. Equivalent circuit of prepared Cu_2O/overlayer photoelectrodes based on EIS study.

Table S2. Flat band potential and carrier density of overlayers estimated from Mott-Schottky analysis.

Sample	V_{fb} (V vs. RHE)	N_A (cm^{-3})
ZnO	0.067	1.18×10^{17}
AZ@AZO	−0.235	9.71×10^{17}
AA@AZO	−0.282	1.58×10^{18}
ZA@AZO	−0.139	1.47×10^{18}
ZZ@AZO	−0.220	1.52×10^{18}
